# Supplementary material for: Smooth Interpolating Curves with Local Control and Monotone Alternating Curvature
Source: Comput Graph Forum. 2022 Oct 6;41(5):25–38. doi: 10.1111/cgf.14600 (PMC9827861; doi:10.1111/cgf.14600)
Supplement: Supplementary file 1 — Supplement Material [file CGF-41-25-s001.zip › Local-Smooth-Interpolating-MonoCurvature/extern/clothoids/submodules/quarticRootsFlocke/docs/api-cpp/program_listing_file_PolynomialRoots-Utils.hh.html]

Program Listing for File PolynomialRoots-Utils.hh — Quartic Roots v1.0.0

### Navigation

- index
- toc
- Quartic Roots »
- Program Listing for File PolynomialRoots-Utils.hh

# Program Listing for File PolynomialRoots-Utils.hh¶

↰ Return to documentation for file (`PolynomialRoots-Utils.hh`)

```
/*--------------------------------------------------------------------------*\
 |                                                                          |
 |  Copyright (C) 2014                                                      |
 |                                                                          |
 |         , __                 , __                                        |
 |        /|/  \               /|/  \                                       |
 |         | __/ _   ,_         | __/ _   ,_                                |
 |         |   \|/  /  |  |   | |   \|/  /  |  |   |                        |
 |         |(__/|__/   |_/ \_/|/|(__/|__/   |_/ \_/|/                       |
 |                           /|                   /|                        |
 |                           \|                   \|                        |
 |                                                                          |
 |      Enrico Bertolazzi                                                   |
 |      Dipartimento di Ingegneria Industriale                              |
 |      Universita` degli Studi di Trento                                   |
 |      email: enrico.bertolazzi@unitn.it                                   |
 |                                                                          |
\*--------------------------------------------------------------------------*/

#ifndef RPOLY_HH
#define RPOLY_HH

#include <utility>
#include <cstdlib>
#include <cmath>
#include <complex>
#include <iostream>
#include <limits>

/*
..
.. N. FLOCKE
.. Algorithm 954: An Accurate and Efficient Cubic and Quartic Equation Solver
.. for Physical Applications
.. ACM TOMS, Vol. 41, No. 4, 2015.
.. DOI: http://dx.doi.org/10.1145/2699468
..
*/

#include <cstdint>

namespace PolynomialRoots {

  typedef double valueType;
  typedef int    indexType;
  typedef std::complex<valueType> complexType;

  #ifndef DOXYGEN_SHOULD_SKIP_THIS

  static int       const bitsValueType = std::numeric_limits<valueType>::digits;
  static valueType const splitFactor   = static_cast<valueType>((std::uint64_t(1)<<(bitsValueType-2))+1); // one extra digit is implicitly 1

  /*
  ||         _   _ _
  ||   _   _| |_(_) |___
  ||  | | | | __| | / __|
  ||  | |_| | |_| | \__ \
  ||   \__,_|\__|_|_|___/
  */
  // - - - - - - - - - - - - - - - - - - - - - - - - - - - - - - - - - - - - - -
  // a + b = x + err
  static
  inline
  void
  TwoSum(
    valueType   a,
    valueType   b,
    valueType & x,
    valueType & err
  ) {
    x = a+b;
    valueType z = x-a;
    err = (a-(x-z))+(b-z);
  }

  static
  inline
  void
  TwoSum(
    complexType   a,
    complexType   b,
    complexType & x,
    complexType & err
  ) {
    valueType s1, e1, s2, e2;
    TwoSum( a.real(), b.real(), s1, e1 );
    TwoSum( a.imag(), b.imag(), s2, e2 );
    x   = complexType(s1,s2);
    err = complexType(e1,e2);
  }

  // a = x + y
  static
  inline
  void
  Split( valueType a, valueType & x, valueType & y ) {
    valueType c = splitFactor*a;
    x = c-(c-a);
    y = a-x;
  }

  // a * b = x + err
  static
  inline
  void
  TwoProduct(
    valueType   a,
    valueType   b,
    valueType & x,
    valueType & err
  ) {
    valueType a1, a2, b1, b2;
    Split( a, a1, a2 );
    Split( b, b1, b2 );
    x   = a*b;
    err = a2*b2-(((x-a1*b1)-a2*b1)-a1*b2);
  }

  static
  inline
  void
  TwoProduct(
    complexType   a,
    complexType   b,
    complexType & p,
    complexType & e,
    complexType & f,
    complexType & g
  ) {
    valueType z1, z2, z3, z4, z5, z6, h1, h2, h3, h4, h5, h6;
    TwoProduct(a.real(), b.real(), z1, h1 );
    TwoProduct(a.imag(), b.imag(), z2, h2 );
    TwoProduct(a.real(), b.imag(), z3, h3 );
    TwoProduct(a.imag(), b.real(), z4, h4 );
    TwoSum(z1, -z2, z5, h5);
    TwoSum(z3, z4, z6, h6);
    p = complexType(z5,z6);
    e = complexType(h1,h3);
    f = complexType(-h2,h4);
    g = complexType(h5,h6);
  }

  #endif

}

#endif
```

### Quick search

### Table of Contents

- Quartic Roots Solver
- C++ API

«
hide menu

menu
sidebar
»

### Navigation

- index
- toc
- Quartic Roots »
- Program Listing for File PolynomialRoots-Utils.hh

© Copyright 2021, Enrico Bertolazzi.
Created using Sphinx 4.2.0.
